# Islet Harvest in Carbon Monoxide-Saturated Medium for Chronic Pancreatitis Patients Undergoing Islet Autotransplantation

**DOI:** 10.1177/0963689719890596

**Published:** 2019-12-30

**Authors:** Hongjun Wang, Wenyu Gou, Charlie Strange, Jingjing Wang, Paul J. Nietert, Colleen Cloud, Stefanie Owzarski, Betsy Shuford, Tara Duke, Louis Luttrell, Aaron Lesher, Klearchos K. Papas, Kevan C. Herold, Pamela Clark, Sahar Usmani-Brown, Jennifer Kitzmann, Craig Crosson, David B. Adams, Katherine A. Morgan

**Affiliations:** 1 Department of Surgery, Medical University of South Carolina, Charleston, SC, USA; 2 Department of Medicine, Medical University of South Carolina, Charleston, SC, USA; 3 Department of Public Health Sciences, Medical University of South Carolina, Charleston, SC, USA; 4 Department of Surgery, University of Arizona, Tucson, AZ, USA; 5 Department of Immunology, Yale University, New Haven, CT, USA

**Keywords:** carbon monoxide, diabetes, insulin independence, chronic pancreatitis, islet transplantation

## Abstract

Stresses encountered during human islet isolation lead to unavoidable β-cell death after transplantation. This reduces the chance of insulin independence in chronic pancreatitis patients undergoing total pancreatectomy and islet autotransplantation. We tested whether harvesting islets in carbon monoxide-saturated solutions is safe and can enhance islet survival and insulin independence after total pancreatectomy and islet autotransplantation. Chronic pancreatitis patients who consented to the study were randomized into carbon monoxide (islets harvested in a carbon monoxide-saturated medium) or control (islets harvested in a normal medium) groups. Islet yield, viability, oxygen consumption rate, β-cell death (measured by unmethylated insulin DNA), and serum cytokine levels were measured during the peri-transplantation period. Adverse events, metabolic phenotypes, and islet function were measured prior and at 6 months post-transplantation. No adverse events directly related to the infusion of carbon monoxide islets were observed. Carbon monoxide islets showed significantly higher viability before transplantation. Subjects receiving carbon monoxide islets had less β-cell death, decreased CCL23, and increased CXCL12 levels at 1 or 3 days post transplantation compared with controls. Three in 10 (30%) of the carbon monoxide subjects and none of the control subjects were insulin independent. This pilot trial showed for the first time that harvesting human islets in carbon monoxide-saturated solutions is safe for total pancreatectomy and islet autotransplantation patients.

## Introduction

Total pancreatectomy and islet autotransplantation (TP-IAT) is a safe and effective approach in the management of intractable pain associated with chronic pancreatitis (CP)^1^. Although quality-of-life parameters are measurably improved in IAT patients, the insulin independence rate after TP-IAT is still low^2–4^. Major hurdles besetting this procedure in CP individuals include the limited number of functional islets available for transplantation from a severely diseased and fibrotic pancreas, and islet cell death caused by stresses encountered after pancreatectomy, during islet isolation, and post-transplantation.

Islet stressors include deprivation of the blood supply after pancreatectomy, exposure to cold enzyme solutions, and pancreatic digestion by enzymatic and mechanical forces^5^. At the end of isolation, islets are transplanted into the liver, a different environment compared with the pancreas, and exposed to the blood, inciting a blood-mediated inflammatory reaction^6,7^. All these procedures cause islet stress. Strategies that produce more “robust” islets that can adequately resist stressors imposed at each step may lead to improved survival of islets, and in the long term, more insulin independence in CP patients undergoing IAT.

Carbon monoxide (CO) has been considered a purely toxic by-product of incomplete combustion processes^8^. Newer evidence suggests that CO at low concentrations acts as a “protective” molecule in cellular processes based on its anti-apoptotic, anti-inflammatory, immunomodulatory, antithrombotic, anti-fibrotic, and vasculo-relaxant effects^9–11^. The therapeutic effects of low-dose CO (<500 parts per million, ppm) have been shown in numerous disease models^12,13^. The United States Food and Drug Administration granted orphan drug designation for inhaled CO for use in the reduction of incidence and severity of delayed graft function in patients undergoing solid organ transplantation. Inhaled CO (100–200 ppm) is being tested in patients with acute respiratory distress syndrome (www.clinicaltrials.gov).

In most studies using CO as a therapeutic agent, inhaled CO has been used. However, translating these results into the clinic might be difficult due to the practical problems associated with scavenging and monitoring levels of a potentially toxic gas. Because CO is soluble in aqueous media (2.3 ml/100 ml at 20°C) and organic solvents, an alternative approach is to dissolve CO in solutions. This approach demonstrated benefits in several animal disease models. For example, a single intra-peritoneal injection of CO-saturated Ringer’s lactate solution ameliorated postoperative ileus in mice^14^. Storage of organs in University of Wisconsin (UW) solution saturated with CO provided significant protection against ischemia/reperfusion injury in the porcine kidney transplantation^15,16^ and the rat liver transplantation models^17^. In a cardiac transplantation model, improved functional recovery was observed after cold storage of the heart with CO-releasing molecule 3^18,19^. In all these studies, no adverse events or abnormal CO-hemoglobin values were observed in recipients. We showed in previous studies that *ex vivo* culture of islets in CO-saturated solution improved survival and function of transplanted islets by suppression of inflammation and β-cell death^20–24^. Inflammation is triggered when the innate immune cells detect injured β cells post-transplantation. Injured cells secrete chemokines that attract leukocytes, migrate to transplanted islets, and increase islet/β-cell death^25^. In mouse studies, CO-treated islet grafts had reduced mRNA expression of chemokines including CXCL5, CXCL6, and CXCL8 compared with control islets at 1 day post transplantation, which may have contributed to improved islet survival after transplantation^24^. This has yet to be tested in humans.

Several indices have been used to determine the viability of islets before and after transplantation. For example, the oxygen consumption rate of cells, which is related to mitochondrial function, has been extensively used to assess viability and health of cells in a variety of fields^26^. In islets, oxygen consumption rate (OCR) divided by cell DNA concentration (OCR/DNA) has been shown as a better indicator of islet viability, and the combination of information on the OCR/DNA (a measure of viability) and OCR dose transplanted (a measure of viable amount of islet tissue transplanted) correlated with transplant outcomes in rodents as well as clinical islet allo- and auto-transplantation^27,28^. One of the indicators to trace or quantify islet cell death post-transplantation is the serum concentration of unmethylated insulin (INS) DNA. Because the insulin gene is uniquely unmethylated in pancreatic β cells, circulating unmethylated *INS* DNA correlates with the amount of β-cell death in mouse models^29^ and humans with CP after TP-IAT^30^.

In the current study, we expanded our findings in animal models to IAT patients and tested whether stress-induced apoptosis of post IAT islets can be minimized by harvesting islets in a CO-rich environment leading to increased islet survival and function. We determined islet cell viability by measuring the OCR of islets and unmethylated *INS* DNA in serum during the peri-transplantation period. The safety and efficacy of this novel intervention has been evaluated until 6 months post IAT. Such studies are needed as there are no proven interventional therapies that reproducibly have improved outcomes of IAT in CP patients.

## Materials and Methods

### Subject Selection

CP patients scheduled for TP-IAT from October 2015 to October 2016 were recruited for this study. All subjects signed informed consent approved by the Medical University of South Carolina (MUSC) Institutional Review Board (IRB). The clinicaltrial.gov registration number is NCT02567240. This study had a Food and Drug Administration exemption. Initial inclusion criteria were 18–69-year-old individuals without prior pancreatic surgery, scheduled for TP-IAT, who were diabetes free according to the American Diabetes Association classification of diabetes, which include: hemoglobin A1C (HbA1c) <6.5%, fasting blood glucose <126 mg/dl and 2-hour post-prandial plasma glucose <200 mg/dl. To increase enrollment, the inclusion criteria was extended to diabetic subjects with IRB approval after the first 13 individuals were enrolled.

### Study Design

The goal of this study was to evaluate the safety and efficacy of harvesting human islets using CO-saturated solutions. This study was designed as a randomized, controlled, double-blind study, with subjects randomized at a 2:1 ratio favoring islets harvested in CO-saturated medium (CO islets) over islets harvested in normal medium (control islets). Post-operative care was not different between groups. The safety of the intervention was measured by adverse events (AEs). Primary efficacy was measured by outcomes of glycemic control as indicated by the area under the curve (AUC) of a mixed meal tolerance test (MMTT) at month 6 post-IAT. Secondary outcomes included post-operative insulin use, insulin independence rate, and quality of life (QOL) as measured by the Short Form 12 (SF-12).

### Production of CO-Saturated Medium

Production of CO-saturated medium has been described previously^24^. In brief, Hanks’ balanced salt solution (Sigma-Aldrich, St. Louis, MO, USA) used for preparation of islet isolation solutions and Viaspan for preserving the islets were bubbled with 1% CO gas for 10 minutes and filtered before use. Total exposure of the islets to CO medium averaged 3–4 hours during the isolation process. All mediums were bubbled fresh, stored in tightly closed bottle without air, and used within 1 hour to avoid release of CO from the medium. The final infusion solution (5% albumin with 70 units/kg of heparin) for subject administration did not have CO bubbling.

### Total Pancreatectomy, Islet Harvest and Transplantation

Total pancreatectomy, islet isolation, and transplantation were performed as described previously^31^. Total islet number, islet viability, islet size index, endotoxin, and mycoplasma levels in the final islet product were measured after islet isolation. Unpurified islets were re-suspended in the infusion solution, and transplanted through the portal vein into the liver. Hepatic pressure before, during, and after islet infusion were recorded. A heparin infusion of 250 IU per hour was given to all patients for 72 hours.

### Monitoring of Adverse Events

Subjects were scheduled to return to MUSC at 1, 2, 3, and 6 months after hospital discharge for study follow-up, and then yearly as a regular TP-IAT patient. All AEs and severe AEs (SAEs) were recorded and classified based on the Clavien-Dindo classification. An independent Data Safety Monitoring Committee reviewed SAEs.

### Measurement of OCR

The OCR was measured in a 1000–3000 islet equivalent number (IEQ) using the MicroOxygen Uptake System, FO/SYSZ-P175 (Instech Laboratories, Plymouth Meeting, PA, USA) as described^32^. The OCR was indexed to the islet DNA content of each sample, measured by fluorospectrophotometry using the Quant-iTTM PicoGreen dsDNA Assay kit (Molecular Probes, Eugen, OR, USA). Fluorescence was read by a Synergy HT microplate reader (BioTek, Winooski, VT, USA). The mean OCR/DNA (nmol O_2_/min·mg DNA) values were calculated as previously described^32^. Samples were measured in triplicate and the mean was used for the final calculation.

### Serum Chemokine Levels

Serum samples were collected from 10 participants (six from the CO group and four from the control group) before pancreatectomy (day 0), and at 1 and 3 days post islet transplantation, allowed to clot, and then centrifuged for 10 min at 1300 × *g* at room temperature, before storage at −80°C. Quantification of 40 human chemokines were performed on serum samples by the magnetic bead-based multiplex immunoassay using the Bio-Plex Pro^TM^ Human Chemokine Panel, 40-Plex kit (Bio-Rad, Hercules, CA, USA), following the manufacturer’s instructions.

### Unmethylated INS DNA

Concentrations of unmethylated and total *INS* DNA were measured in serum samples collected from 10 participants (six from the CO group and four from control group) as described^29^. In brief, DNA was purified from 200 μl of serum using the QIAamp DNA blood kit (Qiagen, Valencia, CA, USA), and treated with bisulfite. Levels of unmethylated and total *INS* DNA was quantified by droplet digital polymerase chain reaction. The ratios of unmethylated divided by total *INS* DNA were calculated.

### Assessment of Clinical Outcome

Diabetes onset and insulin requirements after surgery were measured and compared between subjects receiving CO or control islets. Because all patients were recommended to take insulin post transplantation to reduce the stress of transplanted islets, patients who were completely weaned off insulin post IAT were counted as insulin independent as well. HbA1c, serum C-peptide levels, insulin requirements, and body weight were measured at each follow-up visit for the evaluation of glycemic control. Physical QOL and mental QOL were measured by the SF-12 questionnaire.

### Oral Glucose Tolerance Test and MMTT

An oral glucose tolerance test (OGTT) was performed in all subjects before TP-IAT. Fasting subjects were asked to drink a 75 g glucose solution. Blood samples were collected before and 30, 60, 90, and 120 minutes after glucose ingestion. Blood glucose and serum C-peptide levels were measured using standard methods. MMTT was performed at 6 months post transplantation and blood glucose and serum C-peptide were calculated as described^33^. Blood glucose AUC during an OGTT or MMTT test were calculated using the trapezoidal method.

### Statistical Analysis

Two-tailed independent sample *t* tests were used to compare mean differences between the two groups, and variances were conservatively assumed to be unequal. Difference in insulin independence was compared by Fisher’s exact test. Glucose and C-peptide values after MMTT were compared between groups using general linear mixed models. The difference in unmethylated *INS* DNA was compared by a Mann-Whitney test. All values are presented as mean and standard deviation (SD) unless otherwise specified. A *p* value <0.05 was denoted as statistically significant.

## Results

### Subject Characteristics

In total 16 individuals scheduled for TP-IAT signed informed consent; one failed the initial screening. The other 15 were randomized into CO (*n* = 10) or control (*n* = 5) groups and all had TP-IAT. Characteristics of study participants showed no significant differences in age, body weight, body mass index, HbA1c levels, islet viability, islet size index, and years of CP (Table 1). The first 13 subjects enrolled were diabetes free and the last two subjects were diabetic before surgery, and were randomized into the CO group.

**Table 1.** Baseline Characteristics.

	Average of CTR subjects (*n* = 5)		Average of CO subjects (*n* = 10)		
Characteristics	Mean	SD	Mean	SD	*p*
Age (yr)	49	13.1	44.1	11.8	0.48
Body weight (kg)	81	15.2	77.6	30.8	0.82
BMI (kg/m^2^)	29.2	5.8	26.6	9.8	0.59
HbA1C pre-op (%)	5.7	0.3	6.2	1.4	0.29
Years of CP	7.6	3.7	8.1	7.6	0.86
Islet product weight (g)	8.6	3.0	6.3	5.5	0.32
Total islets infused IEQ	218,545	120,422	138,471	100,036	0.24
IEQ/kg	2,656.9	1,402	2,360	2,420	0.77
Hepatic pressure (mmHg)					
Pre-infusion	11.6	5.0	9.3	4.8	0.43
During infusion	15.5	5.9	12.2	4.1	0.37
Post-infusion	16	5.3	13.3	5.6	0.39

BMI: Body mass index; yr: years; HbA1c: hemoglobin A1c; IEQ: islet equivalent number, SD: standard derivation; CO: all subjects receiving CO islets; CTR: subjects receiving control islets; CP: chronic pancreatitis.

There were no differences between basal line total number of islets transplanted between CO and control subjects. On average, controls (*n* = 5) received 218,545 ± 120,422 IEQ islets compared with the CO group, who received 138,471 ± 100,036 IEQ (*n* = 10, *p* = 0.24 vs. subjects receiving control islets (CTR)). The IEQ per kilogram body weight transplanted (IEQ/kg) between both groups was similar (2657 ± 1402 vs. 2360 ± 2420, control vs. CO (Table 1)).

### AEs

All AEs and SAEs seen in both CO and control individuals have been previously seen in the CP patients undergoing TP-IAT (Table 2). There were four SAEs in four of the 13 CO patients and five SAEs from three control patients. There were numerically more episodes of day 3 transaminitis (three) in the CO group. These regressed without therapy by 14 days. A single event termed hepatic artery thrombosis was defined 2 months after surgery, when the hepatic artery failed to visualize on CT angiography. Because the patient did well for the subsequent 3 years, this event may not be present. Most adverse events resolved within a couple of weeks. Because CO exposure was performed during the islet isolation and there was no CO in the infusion solution, investigators deemed the adverse events were most likely not caused by CO exposure to islets.

**Table 2.** Adverse Events at 6 months.

Subject ID	AE description	Severity	Casual relationship	Action taken	SAE?	SAE reason	Outcome
002-CO	Chyle leak	1	1	2		2	1
004-CO	Transaminitis	3	1	2	Y	–	5
	Biliary leak	1	1	2		2	1
005-CO	Diabetic ketoacidosis	1	1	1	Y	2	1
008-CO	Cellulitis of abdominal wall	2	1	2	Y	2	1
011-CO	Acute cystitis	1	1	2			1
012-CO	Wound dehiscence	1	1	3			1
014-CO	Transaminitis	2	1	2			1
015-CO	Transaminitis	2	1	2			1
	Hepatic artery thrombosis	3	1	2	Y	2	1
003-CTR	GI Hemorrhage	1	1	3			1
006-CTR	Hyperglycemia	3	1	2	Y	2	1
	Jejunostomy-tube obstruction	1	1	3	Y	2	1
010-CTR	Dizziness	2	1	1	Y	2	1
	Rectal bleeding	2	1	2	Y	2	1
	Pulmonary embolism	2	1	2	Y	2	1

AE: adverse events; CO: all subjects receiving CO islets CTR: subjects receiving control islets; SAE: serious AEs.

Severity

Grade 1 = mild

Grade 2 = moderate

Grade 3 = severe

Grade 4 = life threating

Grade 5 = death

Casual relationship

1 = unlikely

2 = possibly

3 = probably

Action taken

1 = none

2 = medication

3 = other

SAE reason

1 = life threatening

2 = required hospitalization

3 = prolonging existing hospitalization

4 = resulting in persistent significant disability or incapacity

5 = congenital anomaly or birth defect

6 = medically significant or important medical condition

7 = death

Outcome

1 = recovered

2 = ongoing

3 = recovered with sequela

4 = fatal

5 = unknown

### Pre-Transplant Islet Viability and Post-Transplant Metabolic Phenotypes

**Figure 1.**
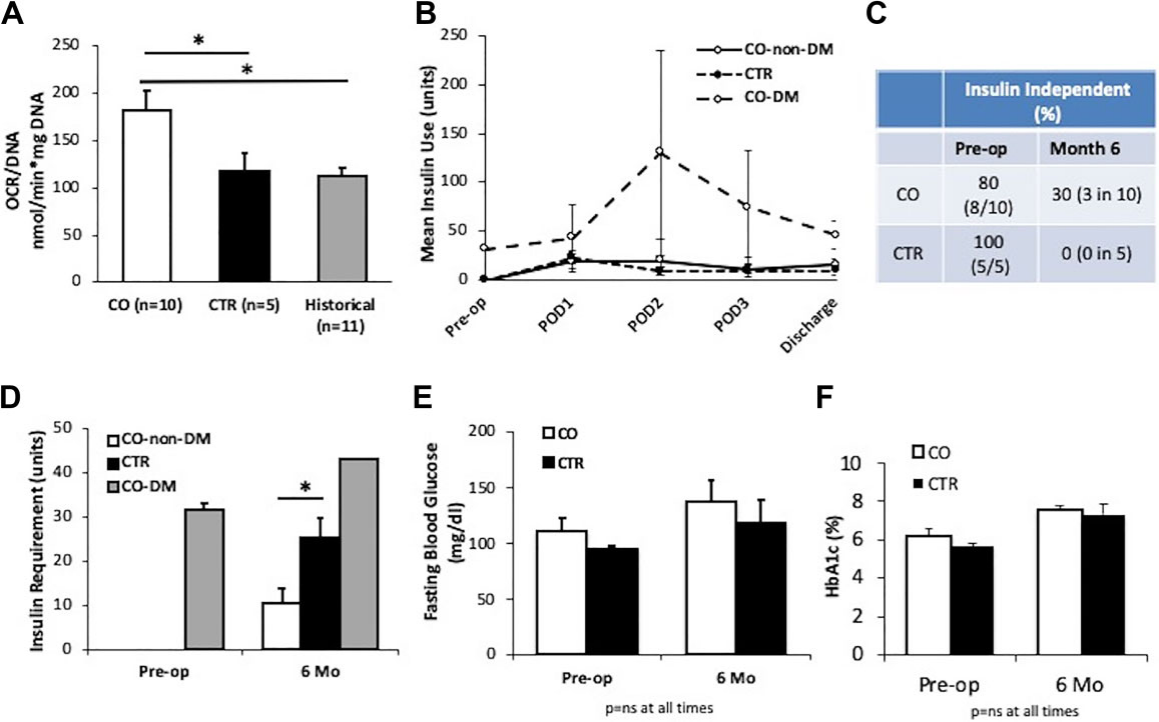
CO islets showed higher oxygen consumption rate (OCR) values and more patients receiving CO islets were insulin independent. (a) OCR/DNA in freshly isolated human islets using CO medium (*n*=10) or normal medium (CTR, *n*=5), and in islets from historical control patients (*n*=11). *p*<0.05, analysis of variance (ANOVA) test followed by Tukey’s multiple-comparisons analysis. (b) Mean insulin needs in non-pre-diabetic CO (CO-non-diabetes mellitus [DM], *n*=8), control (CTR, *n*=5), and pre-diabetic CO (CO-DM, *n*=2) subjects during pre-op, post operation day 1 (POD1), POD2, POD3, and at hospital discharge. (c) Percentage of subjects who were insulin independent before and 6 months after total pancreatectomy and islet autotransplantation (TP-IAT). (d) Mean daily exogenous insulin use in non-pre-diabetic CO (*n*=8), pre-diabetic CO (*n*=2), and control (*n*=5) subjects during pre-op and 6 months after IAT. (e) Fasting blood glucose and (f) mean hemoglobin A1C (HbA1c) levels in CO and CTR subjects during pre-op and 6 months after TP-IAT, *n*=10 for CO subjects, and *n*=5 for CTR. *p<0.05 compared to control. Student’s *t* test assuming unequal variances. CO: all subjects receiving CO islets; CTR: subjects receiving control islets.

We measured OCR/DNA values in freshly isolated CO and control islets. The mean OCR/DNA value of CO islets was 182.5 ± 65.4 (*n* = 10), which was significantly higher than control islets (116.9 ± 46.4, *n* = 5, *p* = 0.04 vs. CO) or islets from historical patients (*n* = 11, *p* = 0.04, Figure 1(a)), suggesting that CO exposure during islet isolation increased islet viability and quality. We measured insulin requirements during the pre-operative period (pre-op), during the hospital stay, and 6 months after transplantation. Two of the diabetic CO subjects required an average of 31.5 units insulin per day pre-op (Figure 1(b)). All TP-IAT patients were given insulin after surgery and before discharge to maintain blood glucose levels at around 100 mg/dl. Non-diabetic CO (*n* = 8) and control (*n* = 5) patients required similar amounts of insulin at day 1, 2, and 3 post-transplantation and at discharge to maintain normoglycemia. The two pre-diabetic CO patients required more insulin to maintain normoglycemia during the peri-transplant period (Figure 1(b)). At 6 months post transplantation, one subject in the CO group did not require any insulin. Two were taking two units of insulin per day at the 6-month visit and weaned off after, therefore were also considered insulin free (Figure 1(c)). The insulin independence rate in the CO group was 37.5% (three in eight subjects who were diabetes free pre-op, *p* = 0.23 vs. control, Fisher’s exact test) or 30% (three in 10 total CO subjects, *p* = 0.51 vs. control). In contrast, all subjects in the control group required insulin at 6 months post TP-IAT, receiving a mean insulin dose of 25.2 ± 4.5 units per day (*n* = 5, Figure 1(d)). Non-pre-diabetic CO subjects required a mean dose of 10.7 ± 3.5 units insulin per day (*n* = 8, *p* = 0.02 vs. control). The two pre-diabetic CO patients required an average of 43.0 ± 21.2 units per day (*n* = 2, Figure 1(d)). There were no differences in fasting blood glucose and HbA1c levels between both groups at 6 months post TP-IAT, although values in both groups were increased compared with pre-op (Figure 1(e) and (f)).

### Change of Islet Function at 6 Months Post-Transplantation

**Figure 2.**
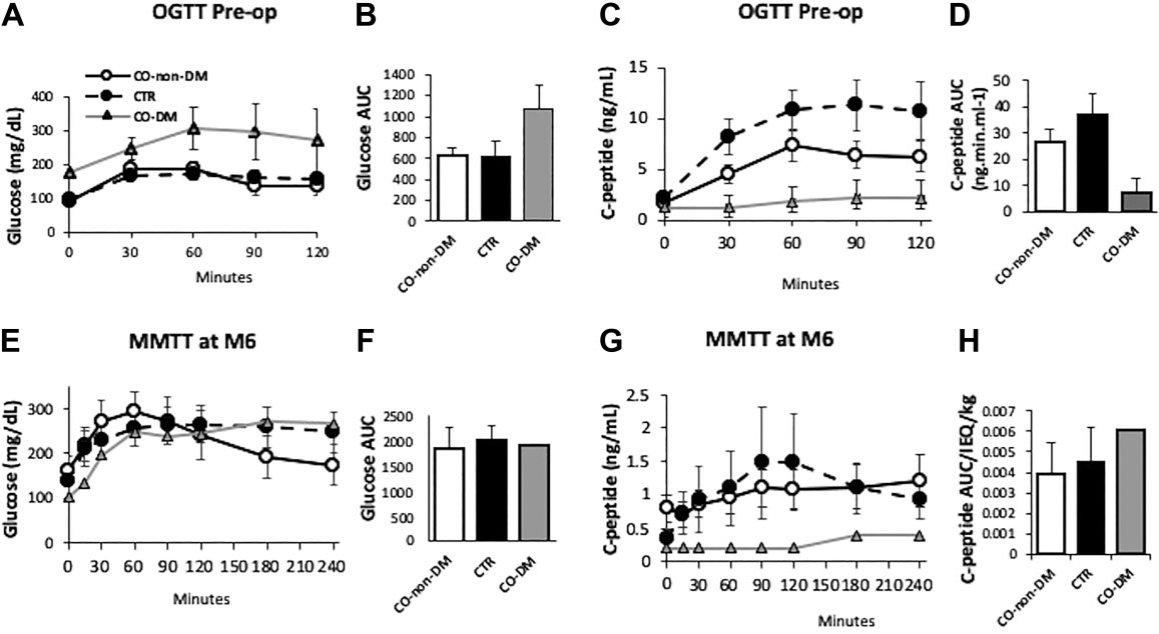
Pre-op oral glucose tolerance test (OGTT) and month 6 mixed meal tolerance test (MMTT). Changes in glucose (a) or C-peptide levels (c) and area under the curve of glucose (b) or C-peptide (d) during the pre-op OGTT tests in pre-diabetes carbon monoxide (CO) (CO-diabetes mellitus [DM], *n*=2), non-pre-diabetic CO (CO-non-DM, *n*=8) and CTR (*n*=5) subjects. Changes in glucose (e) or C-peptide levels (g) and area under the curve of glucose (f) or C-peptide (h) during the MMTT test at month 6 post total pancreatectomy and islet autotransplantation (TP-IAT) in CO-non-DM (*n*=5), CO-DM (*n*=1), and CTR (*n*=5) subjects. CO: all subjects receiving CO islets; CTR: subjects receiving control islets.

Individuals in the control group (*n* = 5) had similar blood glucose levels compared with non-pre-diabetic CO participants (*n* = 8), with the two pre-diabetic CO participants having higher fasting blood glucose levels during a routine OGTT test (Figure 2(a) and (b)). Control subjects (*n* = 5) had higher C-peptide levels compared with both non-pre-diabetic (*n* = 8) and diabetic CO (*n* = 2) participants during the OGTT (Figure 2(c) and (d)). Mean C-peptide AUC was 1.67 times higher in control subjects (*n* = 5) compared with all 10 CO subjects (*p* = 0.06).

MMTT tests were performed in six CO and five control subjects who returned for the test at 6 months post IAT. No difference in mean glucose or C-peptide, or the mean C-peptide AUC divided by IEQ/kg of islets transplanted were observed between the CO and the control subjects (Figure 2(e)-(h)). C-peptide AUC was similar in CTR subjects compared with CO subjects during the MMTT (Figure 2(h)).

### SF-12 QOL

**Figure 3.**
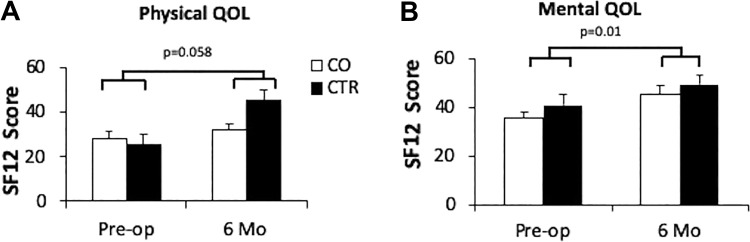
Patients quality of life (QOL) in CO and CTR subjects. Physical (a) and mental (b) QOL of CO (*n*=10) and control (*n*=5) subjects at pre-op and 6 months post-op. Error bars represent standard errors, *p* values are from Student’s *t* test assuming unequal variances. The *p* values were calculated based on QOL at 6 months versus pre-op in all patients. CO: all subjects receiving CO islets; CTR: subjects receiving control islets.

QOL measured by the SF-12 questionnaire was improved at 6 months after surgery compared to pre-op for physical and mental domains in all subjects (Figure 3(a) and (b)). The mean physical QOL increased from 27.67 ± 9.9 to 31.62 ± 8.90 at 6 months in CO, and from 25.5 ± 8.3 to 44.67 ± 11.23 in control subjects (*p* = 0.046 favoring control, Figure 3(a)). The mental health QOL was increased from 33.6 ± 2.7 to 47.0 ± 3.8 in the CO group and 41.0 ± 2.8 to 49.3 ± 4.1 in the control group (Figure 3(b)).

### Serum Chemokine and Unmethylated *INS* DNA Levels during the Peri-Transplant Period

**Figure 4.**
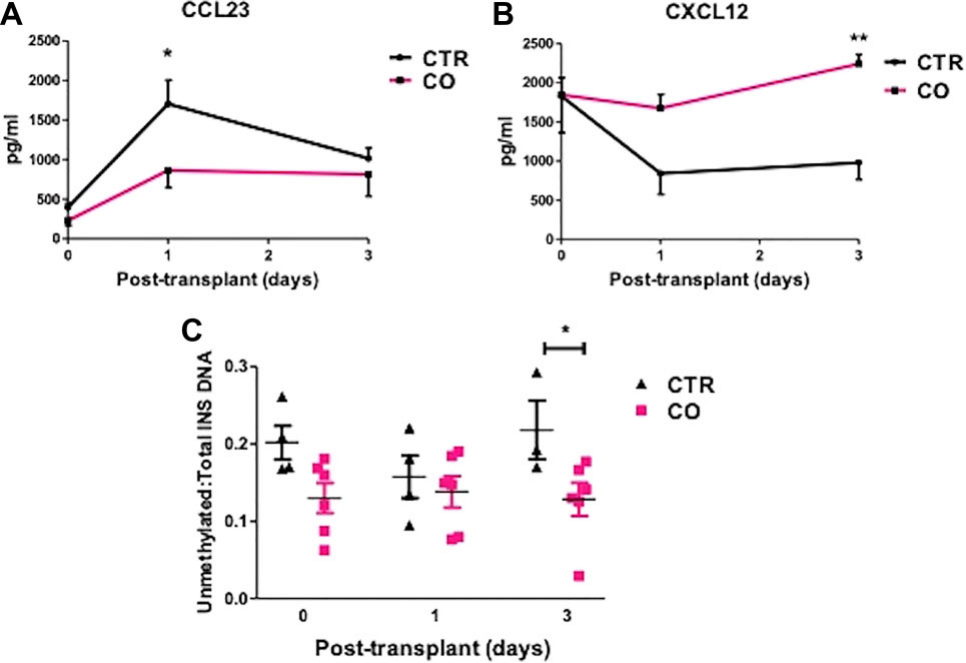
Chemokine and unmethylated insulin DNA concentrations in CO and CTR subjects. Serum concentrations of CCL23 (a), CXCL12 (b), and unmethylated/total insulin DNA (c) in CO or CTR subjects at pre-op, and 1 or 3 days post transplantation. *p<0.05, **p<0.01 vs CTR, *n*=6 in CO and *n*=4 in CTR. CO: all subjects receiving CO islets; CTR: subjects receiving control islets.

Inflammation leads to islet cell death post-transplantation. We measured the serum concentration of cytokines and chemokines in serum collected from CO and control subjects before, at day 1, and day 3 post-IAT. Subjects receiving CO islets had reduced myeloid progenitor inhibitory factor 1 (MPIF1 or CCL23) at day 1 post-transplantation (Figure 4(a)), a cytokine that often increased in subjects with inflammatory diseases^34,35^. Moreover, CO subjects had significantly higher CXCL12, a chemokine that can limit inflammation, at both day 1 and 3 post transplantation (Figure 4(b)). There were no significant differences in levels of other cytokine/chemokines including monocyte chemoattractant protein 1 (MCP 1/CCL2), TNF-α, IL-10, and INF-γ, etc. at both times checked (*Supplemental data, Figure S1*).

To decipher whether reduced chemokine levels are correlated with β-cell death, we measured serum concentrations of unmethylated *INS* DNA, a β-cell death marker. As seen in Figure 4(c), lower unmethylated *INS* DNA was observed in subjects receiving CO islets at 3 days post transplantation, suggesting a trend toward reduced β-cell death in CO islets compared with control islets. Together, these data suggest that CO islets caused less inflammation and survived better after transplantation than control islets.

## Discussion

The exposure of islets or solid organs to CO-saturated solutions protected them from ischemia-reperfusion injuries and cell death in animal models^24,36^. Based on these studies, we performed the first clinical trial evaluating the effects of CO-saturated solutions in preserving islet cell viability and function in CP patients undergoing TP-IAT. This pilot study has demonstrated that CO intervention is safe in this patient population. The documented AEs have been observed in historical patients undergoing TP-IAT. All AEs observed were determined as most likely not related to CO islet transplantation.

A notable trend found in this study is that more patients receiving CO islets were insulin independent at 6 months post transplantation, evident in the observation that 37.5% CO (three in eight) patients who were diabetes free pre-op remained insulin independent at 6 months post TP-IAT. In contrast, none of the five control patients were insulin independent at month 6. The insulin independence rate is also higher than our historical patient population, which was 22% (*n* = 101) at 6 months post IAT^33^. In addition, although the two pre-diabetic CO patients required a higher amount of insulin, non-diabetic CO patients required significantly lower insulin than control patients, which suggests a better function of CO islets.

In this pilot study, subjects with preoperative diabetes were disproportionately enrolled to the CO arm of the study. The CO subjects also had numerically higher baseline HbA1c levels, lower C-peptide AUC values at baseline, and fewer islets infused. The 2:1 randomization led to a better understanding of CO mechanisms, but lower-power results with this trial design. Despite these limitations, CO patients trended toward better islet function and less insulin use at 6 months post TP-IAT.

OGTT, which uses glucose stimulation to provide an abrupt maximal stimulus for insulin release, was performed before TP-IAT in all CP patients as part of the standard of care. MMTT, which uses boost stimulation, provides a slower and more sustained stimulus due to the administration of carbohydrates, protein, and fat and was performed at month 6 post-IAT for subjects who participated in this study to avoid stress on transplanted islets. We realize it is not optimal to compare data from two different tests. Therefore, we compared relative islet function (C-peptide AUC) between the control and the CO groups pre-op and 6 months after IAT. The C-peptide AUCs in controls were 1.6 times higher than CO during the OGTT. In contrast, subjects in both groups showed similar C-peptide AUC (1.1 times difference favoring controls) at 6 months post TP-IAT. Taken together, these data suggest that CO islets did have better survival and function post-transplantation compared to control islets.

Despite missing the primary statistical endpoint of 6-month changes in MMTT outcomes, we remain encouraged by other findings in the study. The difference in insulin independence between the groups was not reflected in the MMTT results because two of the insulin-independent subjects did not return for this MMTT test, whereas one of the preoperative diabetic subjects did. Although it is possible that subjects who did less well did not return for the MMTT test either, these data suggest that CO islets trended toward better survival and function post-transplantation compared to control islets. However, a study with a larger cohort of subject is needed to confirm this hypothesis.

We observed significantly increased OCR/DNA levels in freshly isolated CO islets compared to control islets, supporting that CO exposure enhanced islet viability/quality. We cannot exclude the possibility that higher OCR/DNA in CO islets might have been caused by stresses during islet isolation that reflect a toxicity^37^. However, this likelihood is small because a much higher CO dose had been used and showed profound protection in other disease models^10,16^.

The survival advantage of CO islets was further confirmed *in vivo* by reduced unmethylated *INS* DNA concentration in the serum of CO patients compared to control patients at 3 days post-IAT. However, serum unmethylated *INS* DNA may reach its peak within 3 hours post IAT^30^, therefore, unmethylated *INS* DNA at earlier time points needs to be measured in future clinical trials to confirm the CO effects on islet cell death post transplantation. Nevertheless, it seems reasonable to assume that CO exposure increased islet viability so these islets could better resist stress-induced cell death during the peri-transplant period.

Patients receiving CO islets showed reduced serum levels of CCL23 and increased CXCL12, two chemokines related to inflammation. CCL23 is a newly identified chemokine that can attract monocytes/macrophages, dendritic cells, lymphocytes, and endothelial cells, and can upregulate inflammatory cytokines such as TNF α and MCP-1 in human monocytes. Increased expression of CCL23 was found in patients with inflammatory bowel disease^34^, rheumatoid arthritis, and systemic sclerosis^35^. CXCL12 reduced inflammation in experimental autoimmune encephalomyelitis^38^. It protected β cells from death by promoting islet neovascularization, reduced oxidative stress, and increased cell survival in allogeneic and xenogeneic islet transplantation models^39–42^. Therefore, the significantly decreased CCL23 and increased CXCL12 levels observed in the CO group in our study suggested less inflammation in CO islets post-transplantation. We did not observe notable changes in inflammatory cytokines including MCP-1, IL-8, TNF-α, and INF-γ as often observed in islet grafts after transplantation. A possible explanation is that pro-inflammatory cytokines are more concentrated locally around transplanted islets within a liver and might not be detectable in serum. Because islet sampling in this patient population is not possible, the answer to this question remains unanswered in a human model. Nevertheless, it appears that islets harvested in a CO-rich environment *ex vivo* can protect human islets from cell death and inflammation after transplantation, and ultimately increase long-term islet survival and function.

One limitation that may hinder the clinical application of this approach is that CO is only stable in solution for a few hours before it is released from the medium^43^. Therefore, each medium used must be prepared fresh and used immediately. This limitation was overcome by this study using a simple bubbling procedure. Another strategy is to utilize CO-releasing molecules in lieu of CO gas bubbling to achieve a longer effect^44^. The limitation remains that the beneficial effects of CO-saturated solutions may offer transient islet protection from cell death during the peri-transplant period, but not have longer-term effects. In the future, CO exposure combined with other approaches that are effective in preventing chronic islet cell death could further improve the efficacy of IAT.

This study refined a potential novel approach to improve the efficacy of IAT. We demonstrated the feasibility of enrolling and randomizing study subjects and obtained preliminary estimates of its success by comparing outcomes between the two treatment arms. The data gathered in this study provide key information that can be utilized for the design of a larger multicenter clinical trial. Our preliminary power analysis using an 80% power with a 5% type I error for an intervention that was successful in 3/8 subjects (37%) compared to the average 22% success rate in our large local dataset would require approximately 300 patients to be enrolled in a prospective randomized trial. This would require multicenter efforts.

In conclusion, the results of this study suggest the possibility of procuring human islets in CO-saturated solutions for improving the quality of isolated islets and likely made them more resistant to stresses during islet procurement as well as post-transplant, thus increasing insulin independence in patients undergoing TP-IAT. This study demonstrated a simple, safe, and potentially effective treatment protocol that can be applied to clinical islet transplantation. This treatment option can potentially be applied to allogeneic islet transplantation and other cellular therapies.

## Supplemental Material

Supplemental Material, Figure_S1 - Islet Harvest in Carbon Monoxide-Saturated Medium for Chronic Pancreatitis Patients Undergoing Islet Autotransplantation Click here for additional data file. Supplemental Material, Figure_S1 for Islet Harvest in Carbon Monoxide-Saturated Medium for Chronic Pancreatitis Patients Undergoing Islet Autotransplantation by Hongjun Wang, Wenyu Gou, Charlie Strange, Jingjing Wang, Paul J. Nietert, Colleen Cloud, Stefanie Owzarski, Betsy Shuford, Tara Duke, Louis Luttrell, Aaron Lesher, Klearchos K Papas, Kevan C Herold, Pamela Clark, Sahar Usmani-Brown, Jennifer Kitzmann, Craig Crosson, David B Adams and Katherine A Morgan in Cell Transplantation
