# Supplementary figures and images for: Islet Harvest in Carbon Monoxide-Saturated Medium for Chronic Pancreatitis Patients Undergoing Islet Autotransplantation
Source: Cell Transplant. 2019 Dec 30;28(1 Suppl):25S–36S. doi: 10.1177/0963689719890596 (PMC7016471; doi:10.1177/0963689719890596)

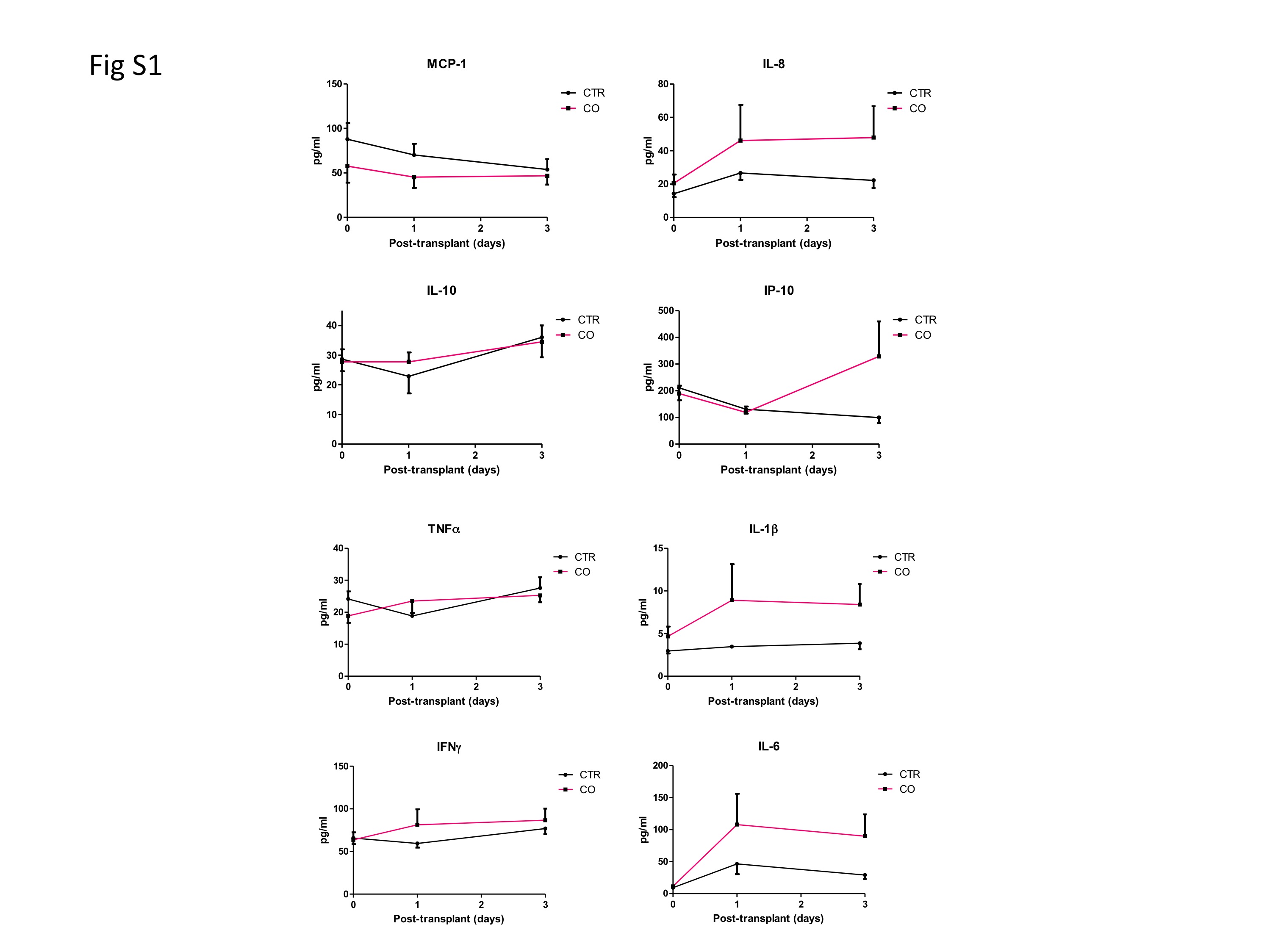

Supplement: Supplemental Material, Figure_S1 - Islet Harvest in Carbon Monoxide-Saturated Medium for Chronic Pancreatitis Patients Undergoing Islet Autotransplantation [file Figure_S1.jpg]
